# High-energy external defibrillation and transcutaneous pacing during MRI: feasibility and safety

**DOI:** 10.1186/s12968-019-0558-z

**Published:** 2019-08-05

**Authors:** Vladimir Shusterman, Denice Hodgson-Zingman, Daniel Thedens, Xiaodong Zhu, Stacy Hoffman, Jessica C. Sieren, Gina M. Morgan, Anthony Faranesh, Barry London

**Affiliations:** 1 PinMed, Inc., Pittsburgh, PA USA; 2 Department of Internal Medicine, The University of Iowa, Iowa City, IA USA; 3 Department of Radiology, The University of Iowa, Iowa City, IA USA; 4 Department of Biological Sciences, The University of Pittsburgh, Pittsburgh, PA USA; 5 National Heart, Lung, and Blood Institute, Bethesda, MD USA

**Keywords:** Magnetic resonance imaging, Cardiovascular magnetic resonance, External defibrillator, External cardiac pacing

## Abstract

**Background:**

Rapid application of external defibrillation, a crucial first-line therapy for ventricular fibrillation and cardiac arrest, is currently unavailable in the setting of magnetic resonance imaging (MRI), raising concerns about patient safety during MRI tests and MRI-guided procedures, particularly in patients with cardiovascular diseases. The objective of this study was to examine the feasibility and safety of defibrillation/pacing for the entire range of clinically useful shock energies inside the MRI bore and during scans, using defibrillation/pacing outside the magnet as a control.

**Methods:**

Experiments were conducted using a commercial defibrillator (LIFEPAK 20, Physio-Control, Redmond, Washington, USA) with a custom high-voltage, twisted-pair cable with two mounted resonant floating radiofrequency traps to reduce emission from the defibrillator and the MRI scanner. A total of 18 high-energy (200-360 J) defibrillation experiments were conducted in six swine on a 1.5 T MRI scanner outside the magnet bore, inside the bore, and during scanning, using adult and pediatric defibrillation pads. Defibrillation was followed by cardiac pacing (with capture) in a subset of two animals. Monitored signals included: high-fidelity temperature (0.01 °C, 10 samples/sec) under the pads and 12-lead electrocardiogram (ECG) using an MRI-compatible ECG system.

**Results:**

Defibrillation/pacing was successful in all experiments. Temperature was higher during defibrillation inside the bore and during scanning compared with outside the bore, but the differences were small (ΔT: 0.5 and 0.7 °C, *p* = 0.01 and 0.04, respectively). During scans, temperature after defibrillation tended to be higher for pediatric vs. adult pads (*p* = 0.08). MR-image quality (signal-to-noise ratio) decreased by ~ 10% when the defibrillator was turned on.

**Conclusions:**

Our study demonstrates the feasibility and safety of in-bore defibrillation for the full range of defibrillation energies used in clinical practice, as well as of transcutaneous cardiac pacing inside the MRI bore. Methods for Improving MR-image quality in the presence of a working defibrillator require further study.

## Background

Magnetic resonance imaging (MRI) is the most rapidly growing imaging modality in clinical medicine, including cardiology [1]. By 2010, the number of MRI scans performed annually in the United States had surpassed 30 million [2]. MRI scans are currently used for the diagnosis and guidance of clinical interventions in such diverse areas as cardiovascular diseases, stroke, trauma, and tumors, as well as for the guidance of neurosurgical and cardiovascular interventions, including cardiac catheterization [3] and electrophysiology (EP) studies [4–10].

However, rapid application of external defibrillation in patients experiencing ventricular fibrillation (VF) or cardiac arrest in the MRI setting is currently impossible, because commercially available external defibrillators cannot be used in the MRI environment [11]. In such situations the patient is removed from the bore, disconnected from the MRI coils and other equipment, and moved to another room, where the defibrillation pads are attached to the patient’s chest and the defibrillator is turned on, passes its internal diagnostic tests, and checks the impedance between the patient’s skin and defibrillation pads. Finally, after passing all these steps, a defibrillation shock can be delivered [11]. However, the defibrillation survival rate decreases rapidly within minutes of VF onset [11, 12]. Due to patient-safety concerns, those at risk for life-threatening arrhythmias, including patients with acute ischemia, severe heart failure, hemodynamic instability, or implanted devices, cannot receive MRI tests or MRI-guided interventions [11, 13–15].

Thus, the absence of an MRI-compatible defibrillator that can provide immediate defibrillation inside the magnet bore impedes the application of MRI tests for high-risk patient populations as well as for guiding cardiovascular procedures (e.g., EP studies) which may require external defibrillation or cardioversion [1]. The feasibility of in-bore defibrillation has been demonstrated in a pilot study for intermediate-energy (200 J) levels only and without an ability to assess electrocardiogram (ECG) changes during defibrillation, because an MRI-compatible ECG was unavailable in that study [11]. The 200 J energy of biphasic defibrillation waveforms is a frequently used energy level for the first discharge, with subsequent increase to 300-360 J if the first shock is unsuccessful, consistent with American Heart Association (AHA) recommendations [16, 17]. However, the feasibility and safety of higher-energy discharges (200-360 J) inside the MRI magnet bore were unknown, as was the feasibility of transcutaneous pacing in an MRI scanner.

The goal of this study was to examine the feasibility and safety of in-bore defibrillation and transcutaneous pacing for the full range of clinically useful energies, including high-energy defibrillation discharges, on a clinical 1.5 T scanner in swine. Similar to a recent study [11], experiments were conducted using a commercial defibrillator (LIFEPAK 20, Physio-Control, Redmond, Washington, USA) with a custom high-voltage, twisted-pair cable and a custom radiofrequency (RF) emissions-filtering setup [18].

To examine the feasibility and safety of high-energy external defibrillation and transcutaneous pacing (with custom cables) in the MRI setting, the experiments were conducted outside the magnet bore, inside the bore, and during active MRI scans with continuous recording of ECG and temperature under the defibrillation electrodes (pads). Importantly, the experiments were performed using both adult and pediatric defibrillation pads.

We used a porcine model, which has been extensively used for studying the physiology of high-energy (360 J) defibrillation discharges [19, 20]. The current density achieved by 360 J discharges in 40-kg animals approaches the AHA’s recommended maximum level for pediatric patients (9-10 J/kg), which has been applied successfully for defibrillation in children with negligible adverse effects [21].

## Methods

All animals were handled in compliance with National Institutes of Health and institutional guidelines according to a protocol that was approved by the Institutional Animal Care and Use Committee of the University of Iowa.

**Fig. 1:**
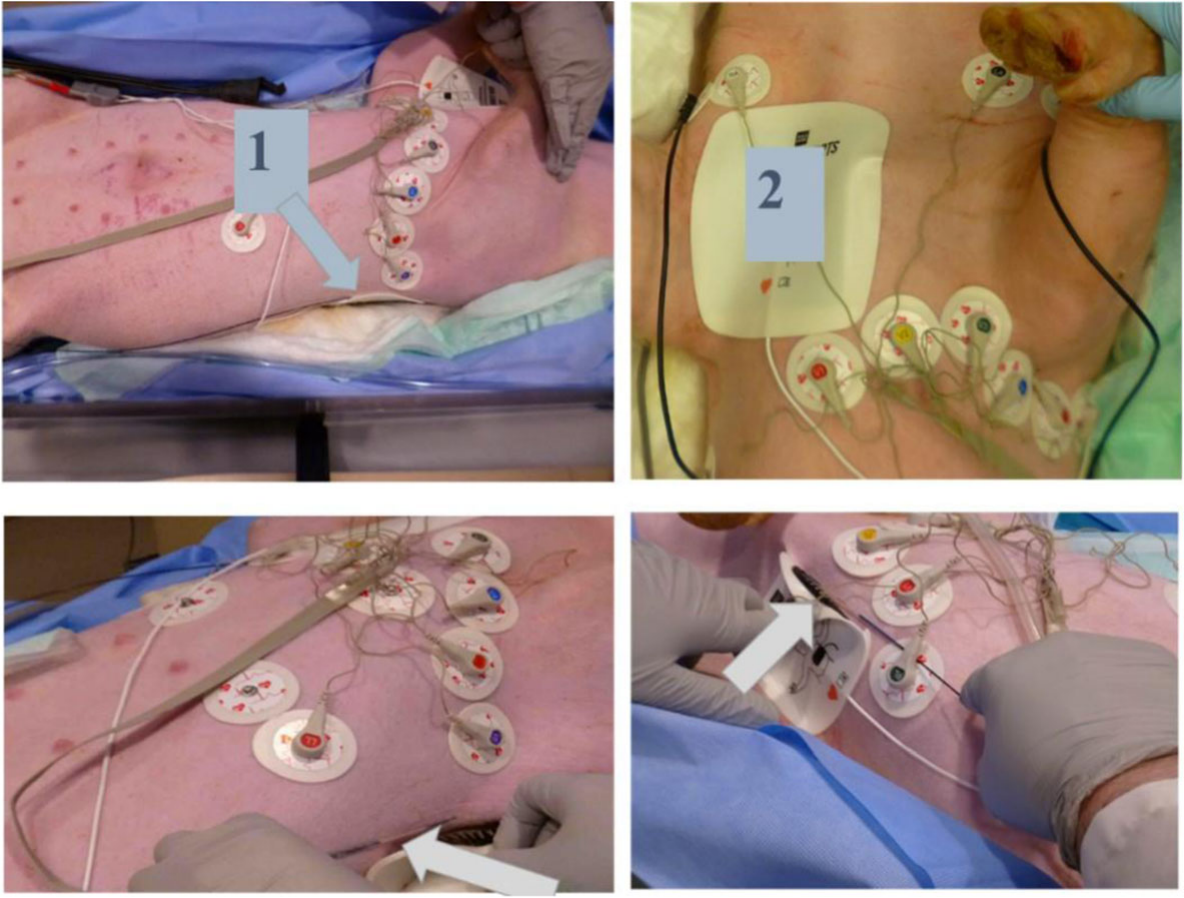
Defibrillator Pad Placement. Defibrillator pads were placed in the left-posterior axillary region (1) and right subclavian region (2). Temperature under the defibrillator pad was recorded continuously using fiber-optic temperature probes (arrows) with 0.01 °C resolution at approximately 10 samples/sec/channel

Eighteen defibrillation experiments (energy: 200-360 J) were conducted in six pigs (five male; weight: 44.7 ± 5.2 kg) on a 1.5 T MRI scanner with a maximum gradient strength of 33 mT/m (Espree, Siemens Healthineers, Erlangen, Germany) at the University of Iowa (Iowa City, Iowa, USA). The animals were anesthetized with isoflurane (0.5–5%) mixed with oxygen via an endotracheal tube. Animals were placed in dorsal recumbency, and adhesive external defibrillator electrodes were applied to the shaved cutaneous surfaces of the lower-left axillary region of the thorax and upper-right subclavian region (standard anterior-lateral placement), as shown in Fig. 1. Commercial heavy-duty polyester belts were used to restrain the animals and withstand the acceleration caused by muscle contraction during defibrillation.

**Fig. 2:**
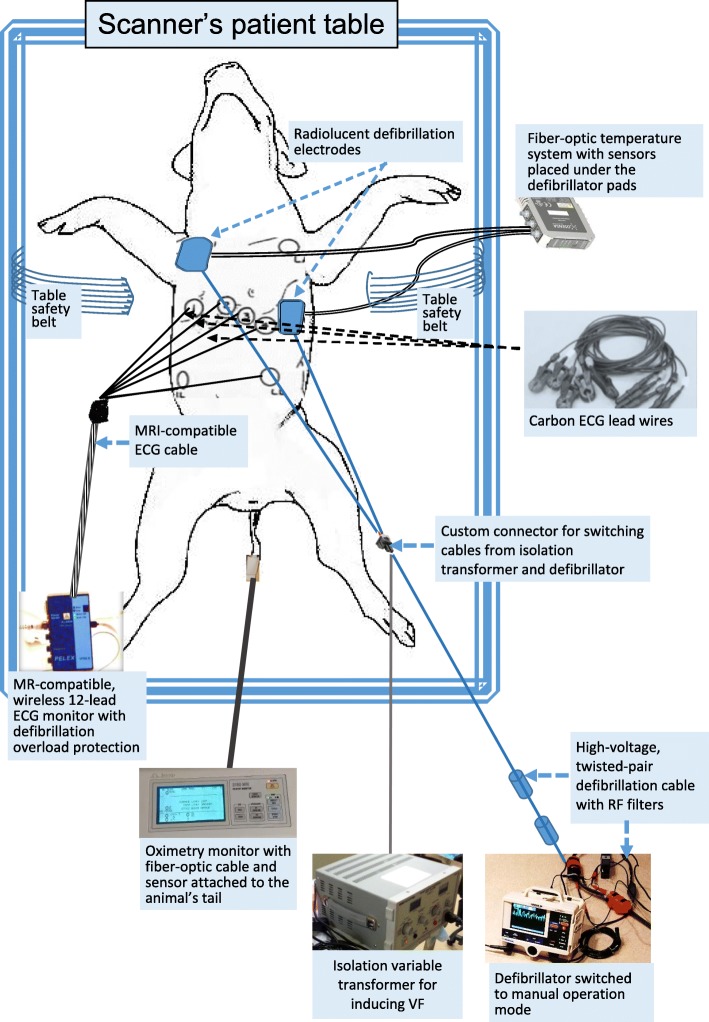
Experimental Setup. The setup included: 1) an MRI-compatible, wireless 12-lead ECG monitor with defibrillation-overload protection and carbon ECG leadwires; 2) a variable isolation transformer, which was used to induce VF; 3) a defibrillator with radiolucent defibrillation pads; 4) a high-fidelity, fiber-optic temperature-monitoring system; and 5) an MRI compatible pulse-oximetry monitor with the sensor placed on the animal’s tail

Experiments were performed using a hospital-grade LIFEPAK 20 defibrillator, which was switched into manual operation mode, with a twisted-pair, high-voltage, low-impedance custom cable (described below), which was connected to a custom system for continuous monitoring of electrical current and voltage produced by the defibrillator for measuring the patient’s transthoracic impedance (Fig. 2). This defibrillator model was selected because it (i) is commonly used during clinical and experimental cardiac EP procedures; (ii) has a wide range of manually programmable options, including pacing and synchronized cardioversion, which provide clinicians with sufficient flexibility in the selection of defibrillation and pacing energies; and (iii) does not generate a strong magnetic-attraction force (as determined using a handheld test magnet) and can be located safely outside the 5-G line.

To test defibrillation safety with the defibrillator placed at various locations and distances from the magnet in the scanner room as well as in the control room, we constructed a 15-m, twisted-pair, high-voltage, multi-stranded, insulated copper cable (outer diameter: 6.5 mm, 19 conducting strands, thermoplastic insulation thickness: 2.2 mm). Resonant non-contact RF traps (“floating baluns”) were placed on the cable to attenuate electromagnetic interference (EMI) generated by the scanner (during imaging) and by the defibrillator during its continuous operation [11]. The chokes (33 × 23 mm) were placed on the cable externally (Fig. 2) near the defibrillator end of the cable; each choke provided approximately 14 dB attenuation at the MRI frequency (64 MHz). Although the defibrillator end of the cable was located outside the 5-G line, we and others have experimentally found that even at such distances, the chokes effectively reduce the impact of EMI on the MR images [22]. At the distal end of the defibrillation cable, we constructed a custom connector for connecting the leads of defibrillation electrodes (pads) and switching between fibrillation and defibrillation cables. Radiolucent defibrillation electrodes (Quik-Combo, Physio-Control) were used to minimize interference (artifact) during MR and X-ray imaging.

Electrical characteristics (current and voltage) were recorded continuously using high-voltage probes. VF was induced through the defibrillator pads by a short (1–2 s) transthoracic application of AC current using a variable isolation transformer (PR57, Sencore, Sioux Falls, South Dakota, USA), as previously described [23]. To determine the threshold for VF induction, the electrical potential energy (voltage) of the AC current was increased in 20 V increments, starting from 30 V, until VF was induced (70 V–100 V). Defibrillation was performed using biphasic, truncated exponential waveforms applied at 200-360 J to test the safety of in-bore defibrillation for the entire range of energies which are utilized in clinical practice [19, 20]. It has been previously shown that pigs provide an appropriate model for testing defibrillation at this energy level [19, 20]. The recording equipment (temperature, current, and voltage) and defibrillator were located on a cart, which was placed in the scanner room (two experiments) or in the control room (four experiments). The MRI-compatible 12-lead ECG monitor was located on the scanner’s patient table.

In each animal, RF-induced heating was tested using a two-dimensional, balanced steady-state free precession (bSSFP) MRI sequence, which is commonly used for cardiac imaging and has a high specific absorption rate (SAR). The sequence was run continuously for six minutes with the heart located at isocenter using the following imaging parameters: time to repeat (TR): 2.8 ms; time to echo (TE): 1.48 ms; flip angle (FA): 70°; acquisition matrix: 256 × 161; field of view (FOV): 33.7 × 45.0 cm; slice width: 6 mm; bandwidth: 1028 Hz/pixel; SAR: 3.6 W/kg. To test for signal-to-noise-ratio (SNR) reduction in the presence of the defibrillation system, we also used a cardiac-gated, two-dimensional, bSSFP sequence with the following parameters: TR: 3.12 ms, TE: 1.56 ms, flip angle: 70°, matrix: 192 × 192, FOV: 500 × 500 cm, slice width: 5 mm, Bandwidth: 1000 Hz/pixel. The SNR was calculated as previously described [11].

To determine the trajectory of the temperature changes during continuous bSSFP scanning, we applied a lumped-capacitance heat-transfer system analysis using the maximum, mean, and minimum temperature changes (Max, Mean, and Min T), as previously described [24, 25]. Because the temperature within the body of a small, conductive temperature sensor changes faster than outside the probe, the probe’s Biot number is small (<< 0.1) [24]. The accuracy of the lumped-capacitance model with Biot number < 0.1 was tested using a single-term (single-lump) exponential model of the form T = a − be^−t/c^, where T denotes temperature registered by the sensor and t is measurement time. The model coefficients were determined using nonlinear least squares; root mean square error (RMSE) < 0.03 and R^2^ > 0.98 were required for the model acceptance [24, 25].

The SNR measurements were conducted with the defibrillator located in the scanner room, outside the 5-G line. SNR was measured when: (i) the defibrillator was switched off, (ii) the defibrillator was switched on while it was powered by its internal battery, and (iii) the defibrillator was switched on while it was powered by the 120 V power line.

All other experiments (which did not include SNR measurements) were conducted with the defibrillator connected to the 120 V power line. The defibrillator was located either in the control room, with the high-voltage defibrillation cable threaded through the penetration panel into the scanner room and the external (floating) RF filters located on both sides of the penetration panel, or in the scanner room, with the RF filters located near the defibrillator end of the cable.

In each animal, three consecutive defibrillation tests were performed: (i) outside the magnet bore (control), (ii) inside the bore, and (iii) during scanning (2D bSSFP with the parameters described above), using adult/pediatric (67%/33%) defibrillation pads (Fig. 1).

Cardiac function was monitored before and after defibrillation using an oximetry sensor. Defibrillation success was determined by restoration of regular pulse-oximetry waveforms and regular QRS complexes on the ECG (rate < 150 bpm) [20]. Defibrillation was followed by transcutaneous, constant-rate (non-demand) cardiac pacing at 60 bpm with increasing pacing current (using monophasic, 20-ms pulses, up to 200 mA until capture was achieved) in a subset of two animals.

**Fig. 3:**
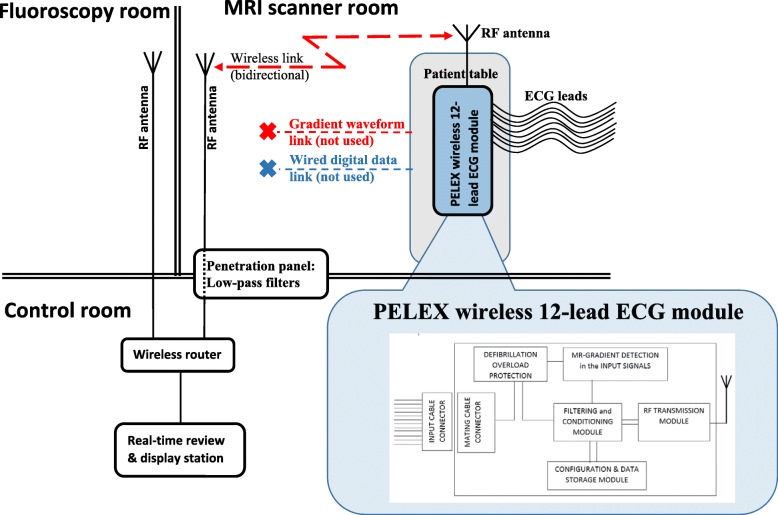
Functional Diagram of the MRI-compatible, Wireless, 12-lead ECG Module with Defibrillation-Overload Protection. The module is located on a patient table and provides both wireless and wired data links. It can move with the table between the MRI and fluoroscopy rooms. Data are transmitted via a bidirectional wireless link and router to a real-time review and display station, which is located in the control room. The module also provides optional gradient waveform and wired digital-data link, which were not used in this study. Inset: block diagram of the 12-lead ECG module. The filtering and conditioning module performs bandpass filtering using either diagnostic (0.05–150 Hz) or monitoring (0.5–50 Hz) frequency range. In addition, the module detects EMI in the input signals (e.g., ECG), using time-domain features of EMI patterns (e.g., derivative and amplitude) and discards the corresponding time sample or inserts an “EMI event” mark to guide the signal-processing algorithm [26, 27]

Monitored signals included 12-lead ECG using an MRI-compatible ECG system (Fig. 3; PinMed, Pittsburgh, Pennsylvania, USA) [26–29] and temperature under the defibrillator pads (Figs. 1 and 2; OSENSA, Burnaby, Vancouver, Canada). The 12-lead, wireless ECG system uses defibrillation-protection circuitry that has undergone and passed mandatory EMI-compatibility testing at an independent EMI-testing facility, which included multiple 5-kV discharges applied to the ECG electrodes during ECG recording to test defibrillator-overload protection, system recovery time after the discharges, reduction in the energy delivered to the patient, operator safety, and other tests specified in the applicable industry standards [30].

Three types of defibrillator electrodes were tested: (i) adult non-radiotransparent (two experiments), (ii) adult radiotransparent (two experiments), and (iii) pediatric [31] radiotransparent (two experiments). Twelve-lead ECG was continuously recorded using radiolucent disposable ECG electrodes placed as close as possible to the Mason-Likar locations (Fig. 1). In addition, oxygen saturation was monitored using an oximetry sensor attached to the animal’s tail (Magnitude 3150 M, Invivo, Gainesville, Florida, USA). Temperature under the defibrillator pads was recorded continuously at 10 Hz/channel using fiber-optic temperature probes with 0.01 °C resolution (Fig. 1). To record temperature changes in different regions of the pads, the temperature probes were placed either in the border zone or inner area of the pads (Fig. 1). A body-surface coil was covered in plastic, placed on the chest, and secured by a table belt.

Because of the small sample size (six animals), we used nonparametric tests, which minimize possible biases for data distributions that are different from normal. The Wilcoxon matched-pairs test was used for comparing temperature during defibrillation outside the bore vs. inside the bore and during MRI scanning. For each dataset, the deviation of the data from normal distribution was assessed using the Kolmogorov-Smirnov test. If the data distribution was normal, t-tests were also performed in addition to nonparametric tests. Because both groups of tests produced consistent results, the data are presented for nonparametric tests only; *p* < 0.05 was considered significant.

## Results

### High-energy defibrillation and transcutaneous pacing

**Fig. 4:**
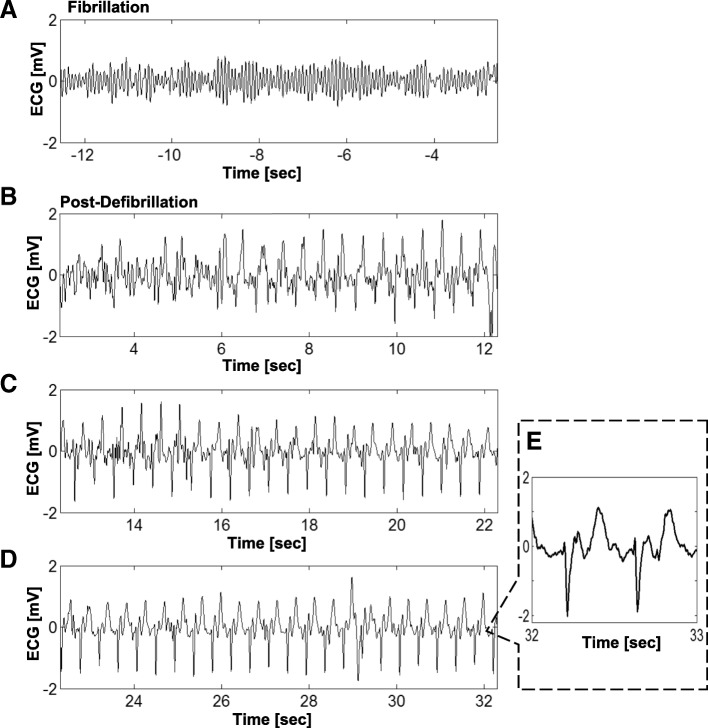
ECG before and after Defibrillation during Active Scanning. ECG in lead V1 before defibrillation (**a**) and after defibrillation (**b**-**d**) during MRI scanning (balanced steady-state free precession sequence; bSSFP) in one of the animals (pig #6). Inset (**e**) shows detailed waveform morphology. Defibrillation was performed at time = 0

Defibrillation was successful in all animals as determined by restoration of regular pulse-oximetry waveforms and regular QRS complexes on the ECG (Fig. 4; see Methods). No differences were observed in the success rate of defibrillation inside the bore compared with outside the bore. The straps that restrained the animals were sufficient to withstand the acceleration caused by muscle contraction during defibrillation; animal motion was minimal, and there was no damage to the animals or the MRI equipment.

**Fig. 5:**
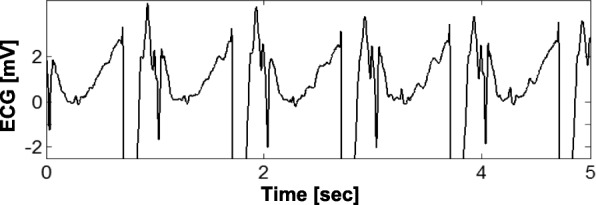
Cardiac Pacing with Ventricular Capture. Sample ECG tracing during transcutaneous, constant-rate (non-demand) pacing at 60 bpm with ventricular capture in the magnet bore in one of the animals (pig #4) using monophasic, 20-ms, 150 mA pulses with rise/fall times ≤1 msec. The pacing was applied in a subset of two animals and produced reliable ventricular capture in both animals

Conduction abnormalities (atrioventricular block), bradycardia, idioventricular rhythm, and escape arrhythmias were observed in four out of six animals after defibrillation due to the repetitive, high-energy discharges used in our study [32]. There was no difference in the prevalence of conduction abnormalities outside the bore (control) vs. inside the bore. The conduction abnormalities were associated with post-resuscitation cardiovascular collapse, which frequently occurs in pigs after < 3 min of VF and subsequent defibrillation [33] and with isoflurane anesthesia [34]. Transcutaneous pacing was performed in the scanner’s magnet bore in a subset of two animals and produced reliable ventricular capture in both animals (Fig. 5). In each animal, the pacing with ventricular capture continued during 3–5 min without damage to the animals or the MRI equipment.

### Temperature changes under defibrillation pads

**Fig. 6:**
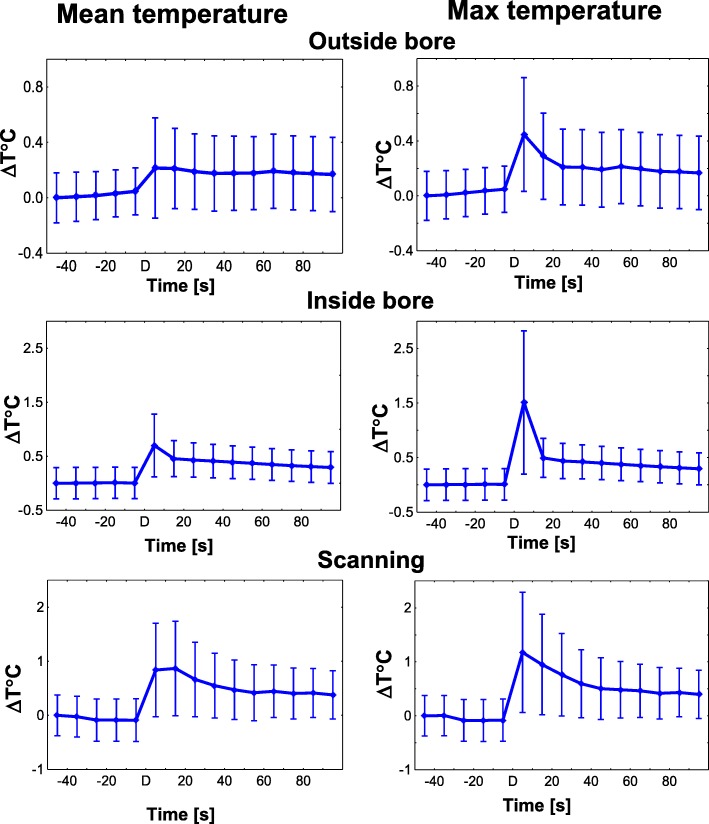
Mean and Max Temperature Changes after Defibrillation outside the Bore, inside the Bore and during Active Scanning in the Studied Animal Group (*n* = 6). The short-term temperature changes after defibrillation were limited to 1.5 °C in five of the six pigs. In the sixth animal, in which defibrillation was performed using pediatric pads, the max temperature increased significantly (> 2 °C) under one of the temperature probes during in-bore defibrillation. Moving the coil (covered in plastic) away from the pad reduced the post-defibrillation changes to approximately 1 °C

Figure 6 shows the dynamics of temperature changes in the entire group of six animals during the period from 50 s before to 100 s after defibrillation (in 10-s intervals).

**Fig. 7:**
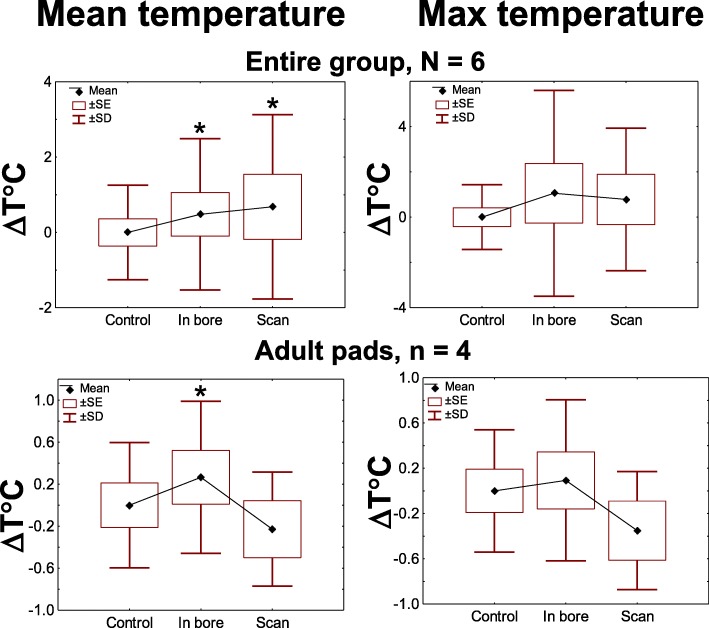
Summary of Post-defibrillation Temperature Changes in the Studied Group. Changes in mean and max temperature (ΔT) 10 s after defibrillation outside the bore (control), inside the bore (in bore), and during bSSFP scan (scan). The temperature changes after defibrillation were slightly larger inside the bore and during scanning compared with outside the bore. Stars indicate significant differences compared with control measurements (*p* < 0.05, Wilcoxon matched pairs test)

Changes in mean and max temperature measured 10 s after defibrillation are shown in Fig. 7 and Table 1. Absolute temperature and its changes (ΔT) after defibrillation were slightly higher inside the bore (T: 37 ± 3 °C, ΔT: 0.5 °C) and during scanning (T: 37 ± 2 °C, ΔT: 0.7 °C) compared with outside the bore (T: 36 ± 1 °C, *p* = 0.01, 0.04, respectively). The differences remained significant when only the four experiments conducted with adult (larger) defibrillation pads were included in the analysis (*n* = 4 animals) (Figs. 6 and 7, Table 2).

**Table 1:** Temperature (°C) increase during defibrillation measured in 10-s averages (*N* = 6)

Temperature	Mean	Std	P (Wilcoxon matched pairs test)
Outside bore	36.35	1.57	
Inside bore	36.88	2.51	0.010
Scanning	37.03	2.45	0.036

**Table 2:** Temperature (°C) increase during defibrillation in experiments conducted with adult defibrillation pads (*n* = 4)

Temperature	Mean	Std	P (Wilcoxon matched pairs test)
Outside bore	35.54	0.60	
Inside bore	35.71	0.70	0.050
Scanning	35.72	0.54	0.465

Temperature increase during defibrillation in five of the six swine was limited to 1.5 °C. In the sixth animal, in which defibrillation was performed using pediatric pads, the temperature increased significantly under one of the two temperature probes during in-bore defibrillation. However, moving the coil (covered in plastic) away from the pad to restore air circulation reduced the temperature increase during in-bore defibrillation to approximately 1 °C.

**Fig. 8:**
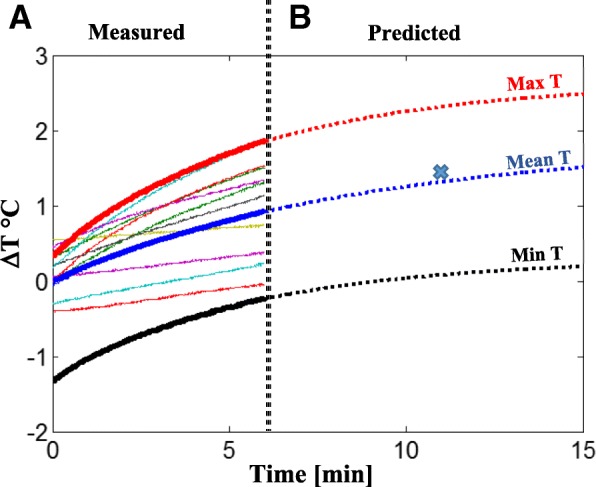
Temperature Changes under Defibrillation Pads during Continuous 2D bSSFP scan. The temperature registered by individual probes during a 6-min scan are shown with baseline temperature subtracted to calculate ΔT°C (**a**); thick lines indicate the maximum (Max T), mean (Mean T), and minimum (Min T) temperature changes. To determine the trajectory of the temperature changes, the lumped-capacitance single-term exponential heat-transfer model was applied in the following form: T = a − be^−t/c^, where T denotes temperature registered by the sensor and t is measurement time [24, 25]. The model satisfied acceptance criteria (RMSE < 0.03 and R^2^ > 0.98), and the model coefficients (a = 2.643; b = 2.295; c = 5.659) were used to predict the Max, Mean, and Min T trajectories beyond the 6-min interval (**b**). Note that the predicted Mean T trajectory is consistent (error: 0.1 °C) with the temperature registered under defibrillator pads after 11 min of similar bSSFP scanning in swine reported by Schmidt et al. (cross) [11]

The temperature changes under the defibrillation pads during continuous six-minute 2D bSSFP scan are shown in Fig. 8. The 6-min trajectories of the measured temperature changes were consistent with those predicted by the single-term lump-capacitance model (Fig. 8; R^2^ = 0.999, RMSE = 0.003) [24]. Furthermore, the predicted trajectory of Mean T was consistent (error = 0.1 °C) with the temperature registered under defibrillator pads after 11 min of continuous bSSFP scanning in similar conditions in swine reported by Schmidt et al. [11]

**Fig. 9:**
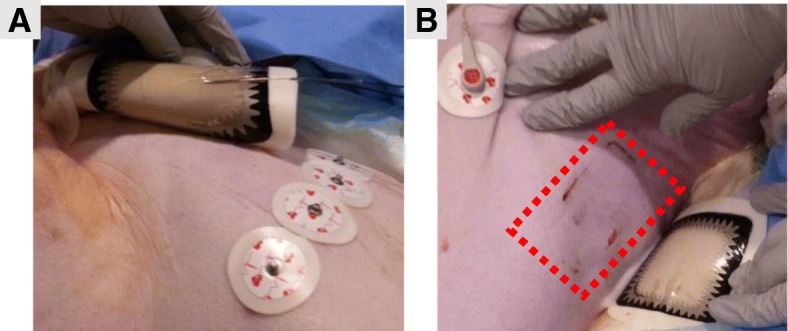
Examples of Skin Changes under the Adult and Pediatric Defibrillator Pads after Defibrillation. **a** After the removal of adult pads, the skin showed minimal or no visible changes. **b** After the removal of pediatric pads, the skin showed either no visible changes or, in one animal, redness and deeper lesions, particularly in the border zone. The affected skin area is demarcated by red dotted line

After the removal of defibrillator pads, the skin was examined for redness and/or burns. After the removal of adult pads, the skin showed minimal or no visible changes (Fig. 9a). Skin changes never exceeded the redness consistent with minor (first-degree) skin burns, which are common after defibrillation events in clinical practice [35]. After the removal of pediatric pads, the skin showed redness and deeper lesions in the border zone in one animal (Fig. 9b).

In addition, the skin was visually inspected under each ECG electrode (after electrode removal) in all animals. No skin changes were observed under the ECG electrodes in any animal.

### MR-image quality in the presence of a defibrillator

**Fig. 10:**
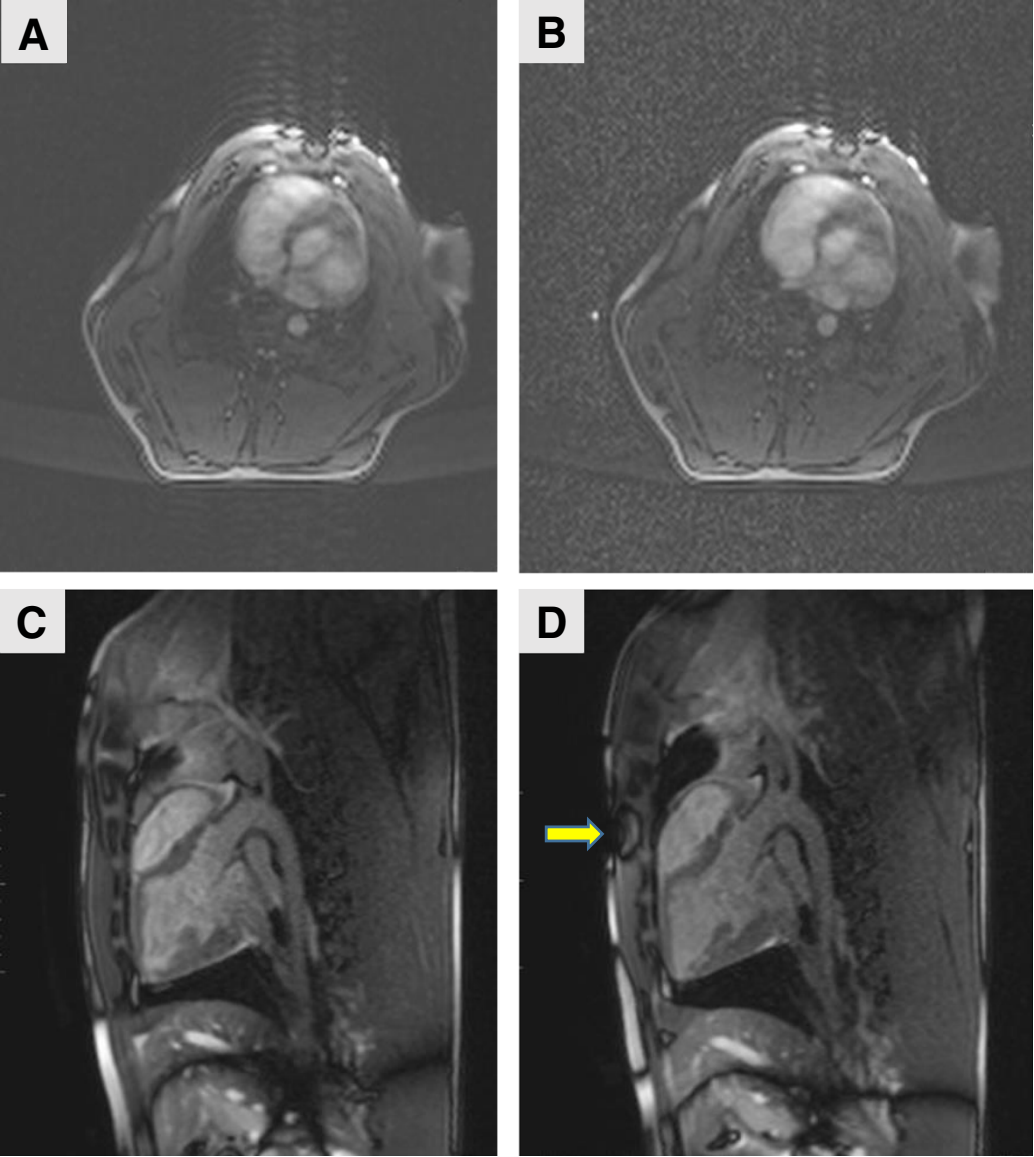
MR Image Quality with the Defibrillator Turned On and Off. bSSFP images were obtained when defibrillator was switched off (**a**) and switched on (**b**). Note the reduction in image quality (due to increased noise level) when the defibrillator was switched on (**b**) compared with when it was switched off (**a**). The images were used to calculate SNR as previously described [11]. There was an approximate 10% reduction in SNR when the defibrillator was switched on. Bottom: image artifact caused by defibrillator pads (**d**, arrow) compared with the same image without the pads (**c**)

The low-amplitude, high-frequency AC current produced by the defibrillator for measuring transthoracic impedance generated a relatively modest reduction in image quality (approximately 10% reduction in SNR). Figure 10a, b shows bSSFP images generated when the defibrillator was switched off and on; the images were used to calculate SNR as previously described [11]. Note the reduction in the image quality when the defibrillator was switched on (Fig. 10b) compared with the image quality when the defibrillator was switched off (Fig. 10a).

The depth of the artifact produced by the defibrillator electrodes was limited to approximately 10–20 mm, and the artifact did not obscure the heart imaging (Fig. 10c and d). No changes were detected in the body-surface coil (noise covariance matrix) before and after each experiment.

## Discussion

Our study shows, for the first time, that high-energy external defibrillation and transcutaneous pacing (with custom cables) are feasible and safe in the MRI setting for the full range of defibrillation energies used in clinical practice [6]. Our study is also the first to document ECG and temperature changes during defibrillation performed in the magnet bore and during an active MRI scan and to compare them with defibrillation outside the bore. It also shows, for the first time, the feasibility and safety of transcutaneous cardiac pacing inside the MRI magnet bore [1].

Defibrillation was successful in all animals inside the magnet bore and during active MRI scans. We measured and compared temperature and 12-lead ECG during high-energy (200-360 J) defibrillation outside the bore vs. inside the bore and during active scans with short duty cycle. Our findings extend the results of a recent report on the safety of in-bore defibrillation using intermediate-level energy (200 J) [11].

### Temperature changes during defibrillation

Potential overheating of the defibrillation pads represents a major concern [6]. To address this issue, the temperature under the defibrillation pads in our study was monitored continuously at a sufficiently high sampling rate (10 samples/sec) to ensure the detection of rapid changes, which occur after discharges. To examine the impact of RF-induced heating, in each animal we measured temperature changes during a 6-min continuous MRI scan using a real-time (bSSFP) sequence with a short duty cycle, which is commonly used in cardiovascular MR tests and which has a high SAR. Our findings are consistent with the recent report by Schmidt et al. [11]; the temperature changes under the defibrillation pads during the continuous 6-min scan (bSSFP sequence) were < 1.5 °C and did not exceed U.S. Food and Drug Administration guidelines [11]. The 6-min trajectories of the measured temperature changes during the continuous bSSFP scanning were consistent with those predicted by the single-term lumped-capacitance heat-transfer model [24], and the theoretically predicted mean temperature change after 11-min bSSFP scanning was similar (within 0.1 °C) to the temperature changes registered under defibrillation pads after similar 11-min bSSFP scanning in swine (1.3 °C at SAR = 3.6 W/kg and 1.4 °C at SAR = 4.4 W/kg, respectively) [11].

Defibrillation inside the bore was accompanied by slightly higher temperatures compared with defibrillation outside the bore, possibly due to limited air circulation in the bore. However, the difference was too small (< 1 °C) to produce any clinically significant effects. Similarly, the small difference between the temperature changes during scanning and the temperature changes inside the bore without an active scan in the subgroup of animals with adult defibrillation pads (Fig. 7) did not reach statistical significance.

### Skin changes under defibrillation pads

Skin changes under the adult pads were either absent or limited to redness and irritation, which are consistent with first-degree burns (Fig. 9a).

Under the pediatric pads, which during defibrillation lead to higher-density electrical currents, we observed deeper lesions primarily in the pad border zone. These lesions were expected, because we applied 360 J discharges to the pediatric pads (in a subset of two animals) to test a worst-case scenario and to confirm the validity of the AHA’s recommendation to limit the use of pediatric pads to children weighing < 22 lbs. (< 10 kg) and a maximum dose of 10 J/kg (resulting in a total energy of 100 J) [21]. The skin changes also could have been exacerbated by the temperature probes, which were placed in the border area and may have increased skin-electrode impedance (Fig. 9b). Furthermore, because we used multiple (3–10) consecutive defibrillation discharges, which were separated by short (several-minute) intervals, their cumulative effect may have increased the probability of skin changes.

The skin changes could have been further exacerbated by the plastic cover of the body-surface coil, which protected the coil but impeded normal air circulation. Indeed, moving the plastic-covered coil away from the pad to restore air circulation reduced the temperature increase during in-bore defibrillation. Further research is warranted to examine skin changes when the coil is not covered by plastic and air circulation is improved.

### ECG changes during defibrillation

Our study compared the effects of defibrillation inside and outside the MRI magnet bore. Defibrillation was successful in all animals, both inside and outside the bore, as manifested by the restoration of regular ECG rhythm. In four out of six animals, the multiple high-energy discharges resulted in conduction abnormalities and subsequent hemodynamic collapse, which frequently occurs in pigs after < 3 min of VF and subsequent defibrillation [23, 33] and can be exacerbated by isoflurane anesthesia [34]. However, there were no differences between the frequency of conduction abnormalities or hemodynamic collapse during defibrillation inside the bore compared with outside the bore.

### Animal movement during defibrillation

Animal movement during defibrillation is primarily determined by strong muscle contraction, with significant acceleration of the limbs, if the animal is not secured to the table [36]. In our experiments, the animals were secured by table straps, which are commonly used during MRI tests in clinical practice. Our results show that securing a subject to the table using standard straps is sufficient to ensure patient safety during in-bore defibrillation.

### Image quality

The 10% image-quality (SNR) reduction in our study was comparable to the 13% reduction observed in the study by Schmidt et al. [6] when the defibrillator was powered by an internal battery. When the defibrillator was powered from a power line, there were no changes in the SNR image quality in our study, whereas Schmidt et al. reported a substantial (31%) decrease. Although we used similar passive, common-mode RF filters (chokes), the filter composition, size, and RF-shielding effectiveness (see Methods for details) may have been different. In addition, the difference could result from multiple factors, including differences in defibrillator model, operating frequencies, positioning of the defibrillation cables relative to the region of interest and imaging plane, and the anatomies of the studied animals. Further research is warranted to identify the primary factors affecting image quality and to develop strategies for mitigating these effects.

The fact that EMI reduction is achieved by placing the chokes near the defibrillator end of the cable, which is located outside the 5-G line, seems counterintuitive. However, our extensive testing showed that this approach effectively reduces the impact of EMI generated by the scanner’s RF emission and by the defibrillator during its continuous operation on the MR image quality by several orders of magnitude [22].

There are several putative mechanisms that could explain these observations:

1. Although the defibrillator was located outside the 5-G line, its cable received the RF-generated EMI from the scanner, because in the near-field area (< 1 wavelength, i.e., ~ 5 m for 64 MHz), the decay of the electric field strength is slower than the decay of the magnetic field (1/r ^2^ vs. 1/r ^3^, respectively, where r is the distance from the EMI source) [37].
2. It is also possible that the placement of chokes close to the defibrillator end of the cable reduced EMI via the standing-wave mechanism, i.e., by reducing the impact of the reflected wave and thus minimizing resonance between waves traveling in opposite directions. This mechanism and the optimal positioning of the RF chokes require further investigation.
3. It is likely that in addition to the choke location, the cable orientation relative to the RF source has some effect on the magnitude of EMI; this also warrants further study.

Further research is also required to identify the primary factors affecting the size of the artifact areas (10–20 mm below the skin surface in our study compared with 6 mm in the study by Schmidt et al. [6]) and to determine an optimal electrode type and material.

## Limitations

There are several limitations associated with temperature monitoring in this pilot study. First, temperatures were measured at a single point under each defibrillation electrode, whereas heat distribution may be concentrated at certain points in the electrode. However, the highest temperature is expected at the border zone, where the electrical impedance of the connection between the electrode and skin surface is usually higher. Indeed, the skin changes were observed only in the border zone (of some pediatric pads), where the temperature probes were located (Fig. 9).

Second, quantitative and histopathological data were not collected from the burn area (Fig. 9), and surface necrosis may not have been immediately apparent. Third, the animal’s core temperature was not monitored in these experiments, and thus heating below the skin surface would have been undetected. Further research is necessary to collect these important safety data. We note, however, that tissue heating from RF radiation during MRI is minimal at the center of a subject’s body and is concentrated at the body’s surface and periphery [38, 39]. Thus, the skin surface was examined carefully under each ECG electrode and under each defibrillation pad in all animals; no skin changes were observed under any ECG electrodes in any animal. There were also no skin changes under the adult defibrillation pads (electrodes). Skin changes were observed in only one animal, in the border zone of pediatric pads, where the temperature probes were located (Fig. 9).

In this pilot study, the heating test was limited to six minutes of continuous scanning with bSSFP, which may not be sufficient to establish thermal safety. Our results, however, are consistent with those reported under defibrillation pads in similar conditions in swine after 11 min of continuous scanning (continuous bSSFP, SAR: 4.4 W/kg) [11].

The temperature of the defibrillation cable was not measured in this pilot study. Although the defibrillation cable did not touch the subject, the cable requires thermal insulation to mitigate the risk of potential heating induced by the RF energy generated by the scanner.

The sample size of animal subjects used in these pilot experiments was small. Further research is warranted to confirm the reproducibility of these pilot results in a larger group and in subjects with electrophysiological abnormalities.

## Conclusions

External defibrillation/pacing is feasible and safe (with cable adaptation) inside the bore of a clinical MRI scanner for the full range of defibrillation energies used in clinical practice. Moreover, external defibrillation is feasible and safe during active MRI scans, opening possibilities for a wide range of MRI-guided EP interventions. Caution is required when defibrillation is performed using pediatric pads, including limiting the defibrillation energy to the AHA’s recommended levels for pediatric pads to avoid skin lesions. Improving MR image quality in the presence of defibrillation is desirable and requires further study.

## Data Availability

The datasets used and/or analyzed during the current study are available from the corresponding author on reasonable request.

## Abbreviations

AHA: American Heart Association
bSSFP: Balanced steady-state free precession
ECG: Electrocardiogram
EMI: Electromagnetic interference
EP: Electrophysiology
FA: Flip angle
FOV: Field of view
MRI: Magnetic resonance imaging
RF: Radiofrequency
RMSE: Root mean square error
SAR: Specific absorption rate
SNR: Signal-to-noise ratio
T: Temperature
TE: Time to echo
TR: Time to repeat
VF: Ventricular fibrillation
